# Integrated Analysis of Single-Cell RNA-Seq and Bulk RNA-Seq Unravels the Molecular Feature of Tumor-Associated Macrophage of Acute Myeloid Leukemia

**DOI:** 10.1155/2024/5539065

**Published:** 2024-01-02

**Authors:** Xin Gao

**Affiliations:** Anhui Medical College, Hefei, China

## Abstract

**Background:**

The association between acute myeloid leukemia (AML) and macrophage remains to be deeply explored.

**Methods:**

Gene expression profiles and clinical variable characteristics of AML patients were collected from TCGA, GEO, and TARGET databases. Consensus clustering was employed to construct the macrophage-related clusters. The macrophage-related index (MRI) was constructed using the LASSO and multivariate Cox analysis. The GSE71014 and TARGET datasets were utilized as external validation sets. Single-cell sequencing data for AML (GSE116256) was adopted to analyze modeled gene expression levels in cells.

**Results:**

Two macrophage-related clusters with different prognostic and immune infiltration characteristics were constructed in AML. Cluster B had a poorer prognosis, more cancer-promoting pathway enrichment, and an immunosuppressive microenvironment. Relied on the MRI, patients of different groups showed different levels of immune infiltration, different mutations, and prognoses. LGALS1 and BCL2A1 may play roles in promoting cancer in AML, while ELANE may have a significant effect on suppressing cancer.

**Conclusion:**

Macrophage-related genes (MRGs) had significant impacts on the occurrence and progression of AML. MRI may better evaluate the prognosis and immune features of AML patients.

## 1. Introduction

AML is one of the most aggressive hematologic malignancies and is a highly heterogeneous leukemia [1, 2]. Currently, chemotherapy and stem cell transplantation are considered the main treatments for patients with AML [3, 4]. However, most AML patients are prone to relapse, and the 5-year survival rate is still less than 30% [5–7]. Therefore, in order to better treat AML patients, it is necessary to screen out biomarkers that can predict prognosis as early as possible.

Tumor-associated macrophages (TAM) are one of the most important immune-related stromal cells in the tumor microenvironment [8]. TAM can not only help tumor cells to modify the microenvironment but also promote tumor proliferation and metastasis and inhibit antitumor immune response [9, 10]. Macrophages mainly include two phenotypes, namely, M1 and M2 macrophages [11]. It is well known that M2 macrophages play a procancer role in tumors [12, 13]. When the M1/M2 polarization balance is broken, TAM will appear to support and stimulate the growth of tumor cells [14]. AML can reestablish monocytes and M*φ*s as phenotypes supporting M2-like leukemia through cell-to-cell contact [15]. Meanwhile, M2 macrophages maintained a close association with poor prognosis in AML, and M1-like macrophages helped prolong patient survival and appeared to inhibit the proleukemic effect [14, 16]. Therefore, macrophage had significant effects on tumor progression, but there was no study to analyze the specific mechanism of macrophage-related genes in AML.

All in all, we comprehensively evaluated the prognostic and immune signatures of MRGs in AML and constructed macrophage-related clusters with different prognostic and immune characteristics. In addition, we employed the Cancer Genome Atlas (TCGA), TARGET, and GSE71014 datasets to construct and validate a macrophage-related prognostic model to evaluate the prognosis of AML. Besides, we further used a single-cell sequencing dataset (GSE116256) to evaluate the expression levels of modeled genes in cells.

## 2. Methods

### 2.1. Data Acquisition and Processing

Table S1 shows the proprietary terms and their corresponding abbreviations. Transcriptome data and clinical data of AML patients were acquired from TCGA and Gene Expression Omnibus (GEO) databases. The GSE71014 [17] and TARGET [18] datasets were utilized as external validation datasets. Immunotherapy data were obtained from the IMvigor210.

### 2.2. Screening of Macrophage-Related Genes

GSE116256 data were derived from Tumor Immune Single-Cell Hub 2 (TISCH2) [19]. In GSE116256 [20], macrophage differential genes were screened based on the adjusted *p* value <0.001 and |logfc|>0.5. Immune-related genes were collected from ImmPort portal (https://www.immport.org/home) and InnateDB (https://www.innatedb.ca/). The intersection genes of immune-related genes and macrophage differential genes were MRGs.

### 2.3. Construction of the Macrophage-Related Clusters

First, the prognostic MRGs were screened by univariate Cox regression analysis. We employed the consensus clustering algorithm to further evaluate the potential mechanism of action of MRGs in AML. Kaplan–Meier (KM) method was employed to assess the survival difference between the macrophage-related clusters. In order to further analyze the differences in biological pathways among the clusters, we screened the differentially expressed genes (DEGs) (|logFC>1| and adj. *p* value <0.001). Gene Ontology (GO) and Kyoto Encyclopedia of Genes and Genomes (KEGG) enrichment analyses were utilized to analyze the underlying mechanisms of DEGs. The gene set variation analysis (GSVA) was a method to further explore the biological signaling pathway. Thus, we performed the GSVA analysis to explore the differences in biological pathways between the macrophage-related clusters.

### 2.4. Establishment of the Macrophage-Related Signature

Depending on the expression profile of prognostic-related MRGs, we further constructed a prognostic model using the least absolute shrinkage and selection operator (LASSO) and multivariate Cox analysis. The MRI was calculated as follows: MRI = Coef A *∗* Exp A + Coef B *∗* Exp B +…Coef N *∗* Exp N. Coef was the coefficient calculated by multivariate Cox analysis and Exp was the expression of MRGs. The KM curve was utilized to evaluate survival differences between high and low MRI groups. The time-dependent receiver operating characteristic (ROC) curve was employed to evaluate the prognostic accuracy of the macrophage-related model.

### 2.5. Evaluation of the Immunogenomic and Mutation Landscape

Tumor microenvironment (TME) may have a significant influence on the process of tumors, so we employed the ESTIMATE algorithm to evaluate the TME score (ImmuneScore, StromalScore, and tumor purity) of AML samples. The single-sample gene set enrichment analysis (ssGSEA) algorithm was constructed to assess the immune function pathway score and immune cell inflation of AML samples. Somatic variant data for AML patients were downloaded from the TCGA database. In the targeted drug therapy analysis, the “pRRophetic” package was adopted to evaluate the half maximum inhibitory concentration (IC50) of 8 common AML chemotherapy drugs.

## 3. Results

### 3.1. Establishment of the Macrophage-Related Clusters and Biological Analysis

**Figure 1:**
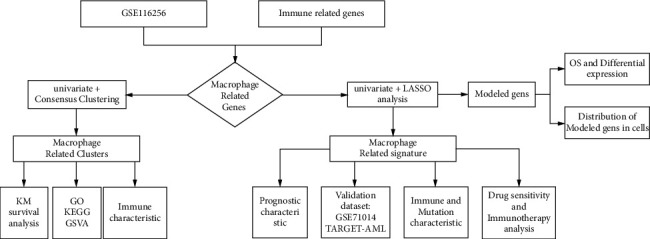
Flowchart of investigation.

**Figure 2:**
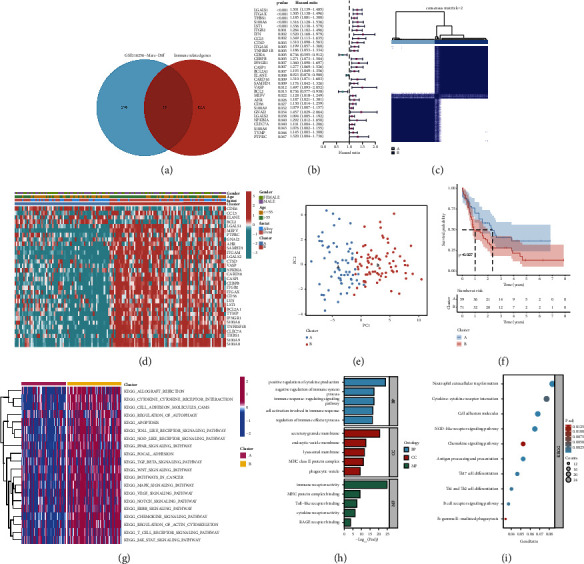
Establishment of macrophage-related clusters and biological analysis: (a) screening of macrophage-related genes, (b) univariate Cox results for macrophage-related genes, (c) different macrophage-related clusters of the TCGA cohort were identified for *k* = 2, (d) distribution of gene expression and clinicopathological variables, (e) sample distribution between classifications, (f) overall survival difference between cluster A and B, (g) GSVA enrichment analysis showed the enrichment distribution of biological pathways in macrophage-related clusters, and (h, i) the results of GO and KEGG enrichment analyses.

Figure 1 shows the research process of this study. A total of 95 MRGs were screened (Figure 2(a)). Thirty-two prognostic-related MRGs were included by univariate Cox regression analysis (Figure 2(b)). We utilized the consensus clustering algorithm to establish 2 disparate macrophage-related clusters according to 32 MRG expressions (Figure 2(c)). Figure 2(d) presents the distribution of the clinical variables and MRG expression. PCA showed the sample distribution in the two macrophage-related clusters (Figure 2(e)). The KM curve revealed that macrophage-related cluster B patients had a worse prognosis (Figure 2(f)). The results of GSVA suggested that tumor-related pathways and immune-related pathways were obviously concentrated in cluster B (Figure 2(g)). GO analysis indicated that DEGs were concentrated in the immune process and cytokine receptor activity (Figure 2(h)). The results of KEGG suggested that DEGs were focused on immune-related pathways and chemokine signaling pathways (Figure 2(i)).

### 3.2. Identification of the Immune Characteristics of Macrophage-Related Clusters

**Figure 3:**
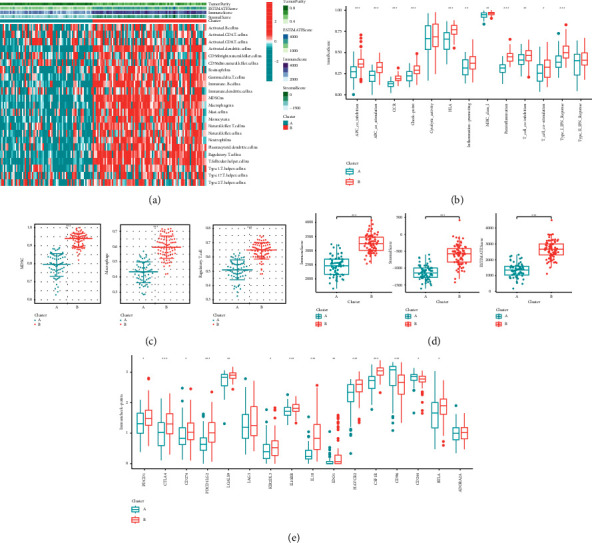
Identification of the immune cell infiltration characteristics of macrophage-related clusters: (a) the distribution of immune cell and TME scores, (b) differential expression of immune function score between macrophage-related clusters, (c) differential expression of immunosuppressive cells (MDSC, macrophage, and regulatory T cell) between macrophage-related clusters, (d) differential expression of TME scores (ImmuneScore, ESTIMATEScore, and StromalScore) between macrophage-related clusters, and (e) differential expression of immunosuppressive checkpoints between macrophage-related clusters.

To further explore the causes of survival differences among macrophage-related clusters, we analyzed the characteristics of immune cell infiltration. The heat map presented the distribution of TME scores and immune cells between macrophage-related clusters (Figure 3(a)). Moreover, multiple immune function pathways were obviously highly expressed in cluster B (Figure 3(b)).  Figure 3(c) shows that immunosuppressive cells (myeloid-derived suppressor cells (MDSC), macrophage, and regulatory T cell) were significantly enriched in cluster B. Besides, TME scores (ImmuneScore, ESTIMATEScore, and StromalScore) and most immunosuppressive checkpoints were highly expressed in cluster B (Figures 3(d) and 3(e)).

### 3.3. Establishment and Validation of the Macrophage-Related Index

**Figure 4:**
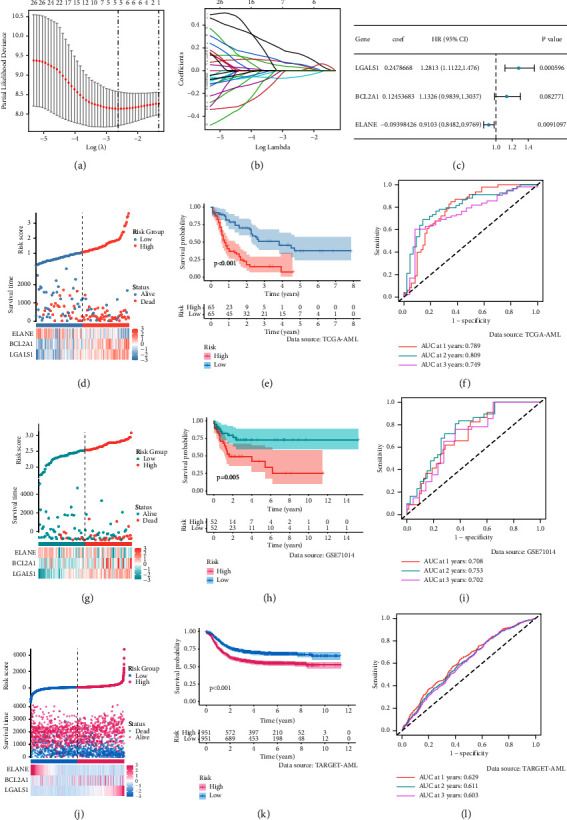
Establishment and validation of the macrophage-related index: (a) LASSO regression of the 5 prognostic macrophage-related genes, (b) LASSO coefficients for 5 prognostic macrophage-related genes, (c) multivariate Cox results for 3 modeled genes, (d, g, and j) the risk curve of each sample reordered by macrophage-related index and the scatter plot of the sample survival overview. D: TCGA, G: GSE71014, J: TARGET, (e, h, and k) KM curve showing the prognostic difference between high and low macrophage-related index groups. E: TCGA, H: GSE71014, K: TARGET, and (f, i, and l) ROC curves about disulfidptosis related signature in 1, 2, and 3 years. F: TCGA, I: GSE71014, L: TARGET.

After the Lasso regression analysis of 32 prognostic MRGs, we screened out 5 prognostic genes (Figures 4(a) and 4(b)). Then, we further used multivariate Cox regression analysis to select three MRGs to construct the model (Figure 4(c)) (MRI = 0.2478668 *∗* LGALS1^Exp^ + 0.12453683 *∗* BCL2A1^Exp^ + (−0.09398426) *∗* ELANE^Exp^). The KM curve and survival status distribution suggested that high MRI was associated with poor prognosis (Figures 4(d) and 4(e)). ROC curves of MRI in 1, 2, and 3 years were 0.789, 0.809, and 0.749 (Figure 4(f)). GSE71014 and TARGET datasets were adopted as external validation datasets to verify the stability of MRI. The KM curve and survival status distribution in the GSE71014 dataset showed that MRI was associated with poor prognosis (Figures 4(g) and 4(h)). ROC curves of MRI in 1, 2, and 3 years in the GSE71014 dataset were 0.708, 0.753, and 0.702 (Figure 4(i)). The same results were obtained in the TARGET group (Figures 4(j)–4(l)).

### 3.4. Identification of the Immunological Characteristics of Macrophage-Related Index

**Figure 5:**
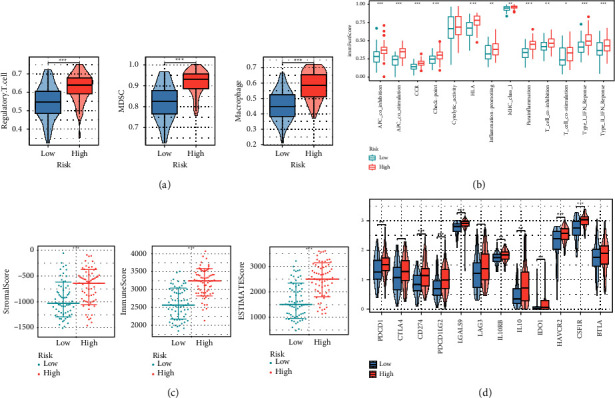
Identification of the immune cell infiltration characteristics of macrophage-related index: (a) differential expression of immunosuppressive cells (MDSC, macrophage, and regulatory T cell) between macrophage-related index, (b) differential expression of imm function score between macrophage-related clusters, (c) differential expression of TME scores (ImmuneScore, ESTIMATEScore, and StromalScore) between macrophage-related index, and (d) differential expression of immunosuppressive checkpoints between macrophage-related index.

Then, we further evaluated the correlation between MRI and immunoinfiltrating cells. Immunosuppressive cells (regulatory T cell, MDSC, and macrophage) were obviously overexpressed in the high MRI group (Figure 5(a)). Immunofunctional pathways such as checkpoint, CCR, and inflammation-promoting were obviously overexpressed in the high MRI group (Figure 5(b)). Besides, the StromalScore, ImmuneScore, and ESTIMATEScore were all significantly overexpressed in the high MRI group (Figure 5(c)). Most immunosuppressive checkpoints were significantly overexpressed in the high MRI group (Figure 5(d)).

### 3.5. Mutation and Immunotherapeutic Responses of the Macrophage-Related Index

**Figure 6:**
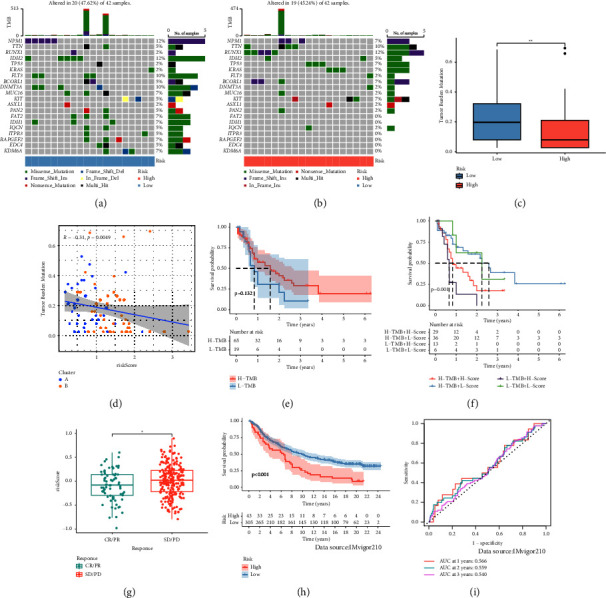
Mutation and immunotherapeutic responses of the macrophage-related index: (a, b) waterfall plots of somatic mutations in high and low macrophage-related index groups, (c) differential expression of TMB between macrophage-related index, (d) correlation analysis of macrophage-related index and TMB, (e) KM survival analysis of TMB groups, (f) survival analysis of distinct groups stratified by both TMB and macrophage-related index, (g) differential expression of risk score between IMvigor210 response groups, (h) KM survival analysis of macrophage-related index groups in the IMvigor210 dataset, and (i) ROC curves of macrophage-related index in 1, 2, and 3 years in the IMvigor210 dataset.

Since tumor mutational burden (TMB) may influence the efficacy of immunotherapy, so we further explored TMB changes in separated MRI groups. The mutation rate was 19/42 (45.24%) in the high MRI group and 20/42 (47.62%) in the low MRI group. In addition, the top 20 genes with mutation rates were the same in the two MRI groups (Figures 6(a) and 6(b)). In addition, TMB was overexpressed in the low MRI group and was negatively associated with MRI (Figures 6(c) and 6(d)). Meanwhile, the low TMB group was linked with poor prognosis (Figure 6(e)). Then, we analyzed the merit of combining MRI with TMB to predict the prognosis of AML. The KM survival curve revealed that L-TMB + H-MRI had the worst prognosis, and H-TMB + L-MRI had the best prognosis (Figure 6(f)). We further found that MRI was significantly overexpressed in the SD/PD group in the IMvigor210 dataset (Figure 6(g)). The KM curve showed that MRI was associated with poor prognosis in the IMvigor210 dataset (Figure 6(h)). ROC curves of MRI in 1, 2, and 3 years in the IMvigor210 dataset were 0.566, 0.559, and 0.540 (Figure 6(i)).

### 3.6. Drug Sensitivity Analysis and Biological Analysis of the Macrophage-Related Index

To further guide clinical strategy development, we analyzed the IC50 differences of 8 chemotherapeutic agents in MRI groups. The results indicated that the IC50 of sorafenib, dasatinib, pazopanib, and bortezomib was higher in the low MRI group (Figures S1A–D) and the IC50 of midostaurin, cytarabine, camptothecin, and axitinib was higher in the high MRI group (Figures S1E–H), suggesting that these 4 drugs (sorafenib, dasatinib, pazopanib, and bortezomib) may be more suitable for patients with higher DMS patients. The results of GSVA enrichment analysis of HALLMARK and KEGG suggested that multiple tumor-associated pathways including mTOR, JAK-STAT, and P53 pathways were enriched in the high MRI group (Figures S2A and B). As a further validation, the results of GO revealed that DEGs were mainly localized to the immune process, MHC protein complex, and cytokine binding (Figure S2C). Simultaneously, KEGG analysis demonstrated that DEGs were focused on multiple immune-related pathways (Figure S2D).

### 3.7. Identification of Prognostic and Expression Characteristics of the Modeled Genes

**Figure 7:**
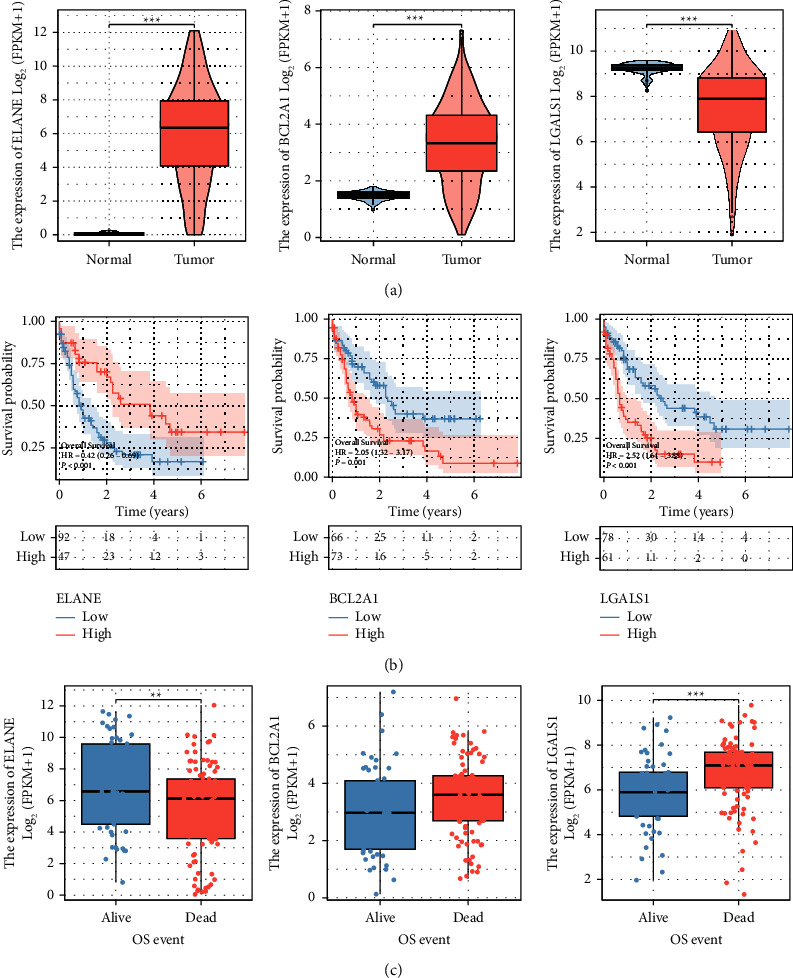
Identification of prognostic and expression characteristics of modeled genes: (a) differential expression of the three modeled genes (ELANE, BCL2A1, and LGALS1) between normal and AML tissues, (b) survival analysis of the three modeled genes (ELANE, BCL2A1, and LGALS1), and (c) expression difference of the three modeled genes (ELANE, BCL2A1, and LGALS1) in Fustat.

**Figure 8:**
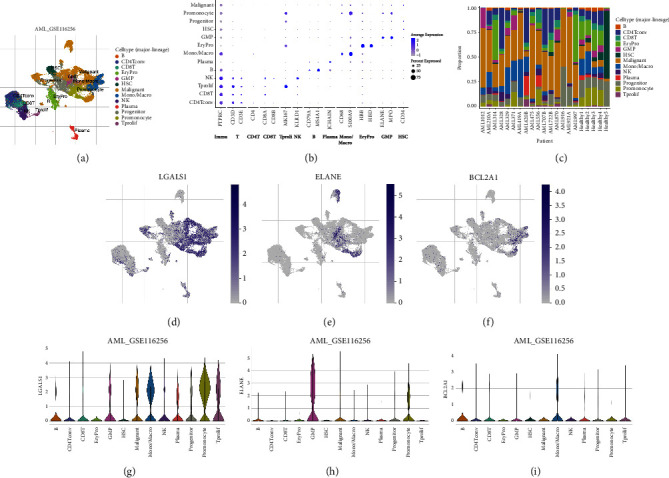
Single-cell sequencing analysis of the three modeled genes: (a) composition and distribution of single cells from GSE116256, (b) the expression of typical marker genes in different cells, (c) composition and distribution of distinct cells in each AML sample, and (d–i) the distribution and amount of three modeled genes (ELANE, BCL2A1, and LGALS1) expression in distinct cells.

Figure 7(a) showed that among the three modeled genes, ELANE and BCL2A1 were significantly overexpressed in tumors, while LGALS1 was suppressed (Figure 7(a)). The KM survival curve indicated that patients with high BCL2A1 and high LGALS1 were associated with poor prognosis, while those with high ELANE had the opposite prognosis (Figure 7(b)). Besides, ELANE expression was low and BCL2A1 and LGALS1 expressions were high in dead patients (Figure 7(c)). The expression distribution of multiple cell subgroups in GSE116256 is shown in Figure 8(a). The cells were classified into different cell lines and labeled with the expression of typical marker genes as shown in Figure 8(b). The proportion of multiple cell subgroups in each GSE116256 patient is shown in Figure 8(c). LGALS1 was highly enriched in malignant, mono/macro, and promonocytes. ELANE was highly enriched in malignant, GMP, and promonocytes. BCL2A1 was highly focused on mono/macro (Figures 8(d)–8(f)). LGALS1 was obviously highly expressed in malignant, mono/macro, and promonocytes (Figure 8(g)). ELANE was significantly highly expressed in GMP (Figure 8(h)). BCL2A1 was highly expressed in mono/macro (Figure 8(i)).

## 4. Discussion

AML is an aggressive myeloid malignancy, and most patients exhibit unsatisfactory prognostic outcomes [21]. With the deepening of research, stem cell transplantation and chemotherapy are of great help to AML patients [21]. However, due to the high recurrence rate of AML, the prognostic survival rate is still not satisfactory [22, 23]. Macrophages play important roles in the immune microenvironment and are widely involved in a variety of tumor development processes [24, 25]. Therefore, we further analyzed the molecular function of MRGs in AML based on their expression profiles, which may help develop more suitable treatment plans and ultimately improve the prognosis of AML patients.

TAM is an important component of TME and plays a complex role in tumor progression [26, 27]. In general, macrophages rely on antigen presentation and secretion of signaling molecules to regulate immunity [27, 28]. However, in tumors, macrophages can be induced into the M1 type with antitumor effects or the M2 type with the induction of anti-inflammatory factors [29]. At the same time, the diversity of TAM in the process of tumor progression also has a certain influence on the efficacy of immunotherapy [30]. Accumulating evidence suggests that TAM is a key mechanism in leukemogenesis and chemoresistance, which has made it an attractive therapeutic target for recent studies. However, the specific mechanism and prognostic model of TAM in AML have not been extensively studied. Thus, in our study, we constructed two macrophage-related clusters with different prognoses. On the one hand, due to the enrichment of immunosuppressive cells and immunosuppressive checkpoints in cluster B, an immunosuppressive microenvironment was formed to facilitate tumor progression. On the other hand, procancer pathways were significantly enriched in cluster B. Besides, the macrophage-related prognostic model was constructed, and the accuracy and stability of the model were verified by external datasets. In TME, invasive immune cells have a significant impact on tumor progression and are extremely significant therapeutic targets [31, 33]. The poor prognosis of the high MRI group may be due to the enrichment of cancer-promoting pathways and immunosuppressive microenvironment. In addition, Wang et al. constructed a prediction model in glioma using 9 TAMs, and the risk score was significantly correlated with patient prognosis and tumor microenvironment [32]. At the same time, Liu et al. established a risk model based on 10 TAMs in head and neck squamous cell carcinoma (HNSCC) and a nomogram that can be used to predict long-term clinical survival [33]. However, developing the best individual treatment plan is a challenge for physicians. In our study, we used the IMvigor210 data to analyze the differences in immunotherapy in the MRI group. MRI expression was significantly low in the CR/PR group, and the prognosis was poor in the high MRI group in the IMvigor210 dataset. In addition, the high MRI group was more sensitive to sorafenib, dasatinib, pazopanib, and bortezomib. These results suggest that the macrophage-related prognostic model may be employed as an important indicator to evaluate the response of AML patients to targeted therapy and immunotherapy and contribute to the formulation and development of personalized treatment for AML patients.

LGALS1 belongs to the galactose lectin family and participates in the construction of the immunosuppressive microenvironment and the regulation of multiple signaling pathways, especially the carcinogenic pathway [34, 35]. LGALS1 has been found to be involved in regulating immunosuppressive microenvironments to regulate tumor progression in many human cancers [36–38]. For example, in glioma, downregulation of LGALS1 inhibited immunosuppressive factors and reshaped the glioma immunosuppressive microenvironment by downregulating M2 macrophages and MDSCs [39]. BCLA2 is an important cell death regulatory factor that controls the release of cytochrome C from mitochondria in the endogenous apoptotic pathway. Research has shown that BCL2A1 is significantly overexpressed in various tumors, including hematological malignancies and solid tumors [40–42]. For example, in a mouse model of MYC-driven leukemia, BCL2A1 can cooperate with MYC to accelerate leukemogenesis [43]. ELANE is one of the key components that take part in the control of the innate immune system, and it participates in the regulation of tumor progression through various mechanisms. Cui et al. showed that ELANE can selectively kill a variety of cancer cells, suggesting a promising anticancer strategy [44]. Besides, in radiation-induced lung cancer, ELANE promotes polarization of M2 macrophages by downregulating PTEN, thus promoting cell proliferation, migration, and invasion *in vitro* [45]. Our study found that LGALS1 and BCLA2 played a cancer-promoting role in AML and were highly expressed in macrophages.

In our study, we first analyzed the prognostic and immune role of MRGs in AML, which may help guide clinical treatment. Although we have extensively analyzed the possible carcinogenic function of MRGs in AML and obtained some reliable results, there were still some deficiencies that needed to be addressed. First, we only used public data to construct and retrospectively validate our findings. Therefore, prospective studies were critical to evaluate clinical efficacy in patients with AML. Second, further biological studies were needed to confirm our findings.

## 5. Conclusion

In brief, we classified AML patients into two macrophage-related clusters with different prognoses and immune cell infiltration characteristics. Moreover, the macrophage-related prognostic model was constructed in AML patients, which may be a marker to predict the prognosis and immune response of AML patients.

## Data Availability

The data that support the findings of this study are openly available in TCGA, GEO, TISCH2, and TARGET datasets.

## Supplementary Materials

Supplementary Materials Figure S1: drug sensitivity analysis of the macrophage-related index. Sensitivity analysis for sorafenib (A), dasatinib (B), pazopanib (C), bortezomib (D), midostaurin (E), cytarabine (F), camptothecin (G), and axitinib (H) in patients between low and high macrophage-related index groups. Figure S2: biological analysis of macrophage-related index: (A, B) the results of GSVA enrichment analysis of Hallmark (A) and KEGG (B), (C) representative enriched GO terms of DEGs in macrophage-related index groups, (D) representative enriched KEGG terms of DEGs in macrophage-related index groups. Table S1 (abbreviation table): proprietary terms and their corresponding abbreviations.
