# Supplementary material for: Integrated Analysis of Single-Cell RNA-Seq and Bulk RNA-Seq Unravels the Molecular Feature of Tumor-Associated Macrophage of Acute Myeloid Leukemia
Source: Genet Res (Camb). 2024 Jan 2;2024:5539065. doi: 10.1155/2024/5539065 (PMC10776189; doi:10.1155/2024/5539065)
Supplement: Supplementary Materials — Figure S1: drug sensitivity analysis of the macrophage-related index. Sensitivity analysis for sorafenib (A), dasatinib (B), pazopanib (C), bortezomib (D), midostaurin (E), cytarabine (F), camptothecin (G), and axitinib (H) in patients between low and high macrophage-related index groups. Figure S2: biological analysis of macrophage-related index: (A, B) the results of GSVA enrichment analysis of Hallmark (A) and KEGG (B), (C) representative enriched GO terms of DEGs in macrophage-related index groups, (D) representative enriched KEGG terms of DEGs in macrophage-related index groups. Table S1 (abbreviation table): proprietary terms and their corresponding abbreviations. [file 5539065.f1.zip › Supplementary Caption--5539065.docx]

***Supplementary Files***

Figure S1 Drug sensitivity analysis of the Macrophage-Related Index

Sensitivity analysis for Sorafenib (A), Dasatinib (B), Pazopanib (C), Bortezomib (D), Midostaurin (E), Cytarabine (F), Camptothecin (G) and Axitinib (H) in patients between low and high macrophage-related index groups.

Figure S2 Biological analysis of Macrophage-Related Index

(A, B) The results of GSVA enrichment analysis of HALLMARK (A) and KEGG (B); (C) Representative enriched GO terms of DEGs in macrophage-related index groups; (D) Representative enriched KEGG terms of DEGs in macrophage-related index groups.

Table S1 Abbreviation table

Proprietary terms and their corresponding abbreviations
