# Supplementary material for: Integrated Analysis of Single-Cell RNA-Seq and Bulk RNA-Seq Unravels the Molecular Feature of Tumor-Associated Macrophage of Acute Myeloid Leukemia
Source: Genet Res (Camb). 2024 Jan 2;2024:5539065. doi: 10.1155/2024/5539065 (PMC10776189; doi:10.1155/2024/5539065)
Supplement: Supplementary Materials — Figure S1: drug sensitivity analysis of the macrophage-related index. Sensitivity analysis for sorafenib (A), dasatinib (B), pazopanib (C), bortezomib (D), midostaurin (E), cytarabine (F), camptothecin (G), and axitinib (H) in patients between low and high macrophage-related index groups. Figure S2: biological analysis of macrophage-related index: (A, B) the results of GSVA enrichment analysis of Hallmark (A) and KEGG (B), (C) representative enriched GO terms of DEGs in macrophage-related index groups, (D) representative enriched KEGG terms of DEGs in macrophage-related index groups. Table S1 (abbreviation table): proprietary terms and their corresponding abbreviations. [file 5539065.f1.zip › Table S1.docx]

| Acute myeloid leukemia (AML) |
| --- |
| Differentially expressed genes (DEGs) |
| Gene Expression Omnibus (GEO) |
| Gene Ontology (GO) |
| Gene set variation analysis (GSVA) |
| Half maximum inhibitory concentration (IC50) |
| Kaplan-Meier (KM) |
| Kyoto Encyclopedia of Genes and Genomes (KEGG) |
| least absolute shrinkage and selector operation (LASSO) |
| Macrophage-related genes (MRGs) |
| Macrophage-related index (MRI) |
| Myeloid-derived suppressor cells (MDSC) |
| Single sample Gene Set Enrichment Analysis (ssGSEA) |
| The Cancer Genome Atlas (TCGA) |
| Tumor associated macrophages (TAM) |
| Tumor Immune Single-cell Hub 2 (TISCH2) |
| Tumor microenvironment (TME) |
