# Supplementary figures and images for: Integrated Analysis of Single-Cell RNA-Seq and Bulk RNA-Seq Unravels the Molecular Feature of Tumor-Associated Macrophage of Acute Myeloid Leukemia
Source: Genet Res (Camb). 2024 Jan 2;2024:5539065. doi: 10.1155/2024/5539065 (PMC10776189; doi:10.1155/2024/5539065)

A

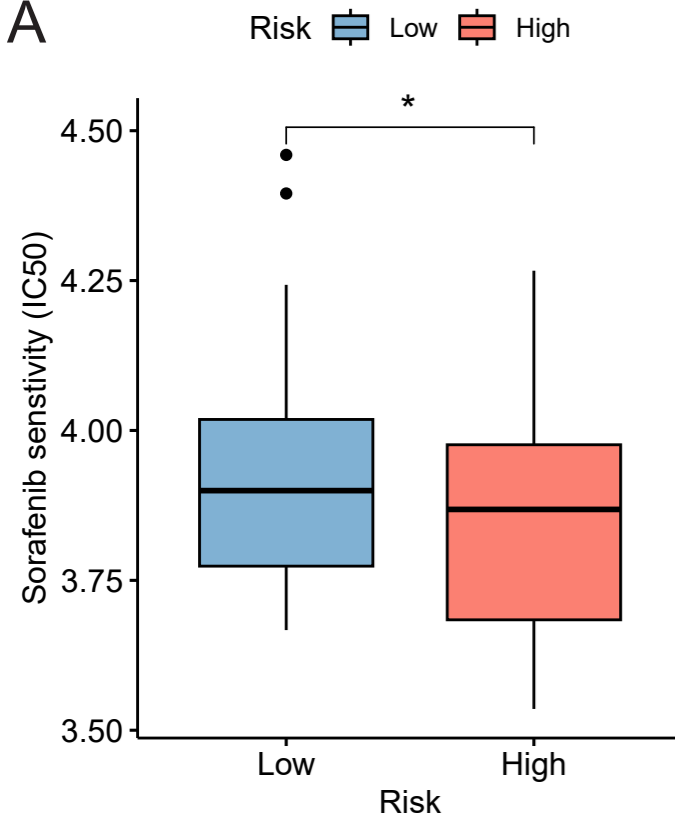

B

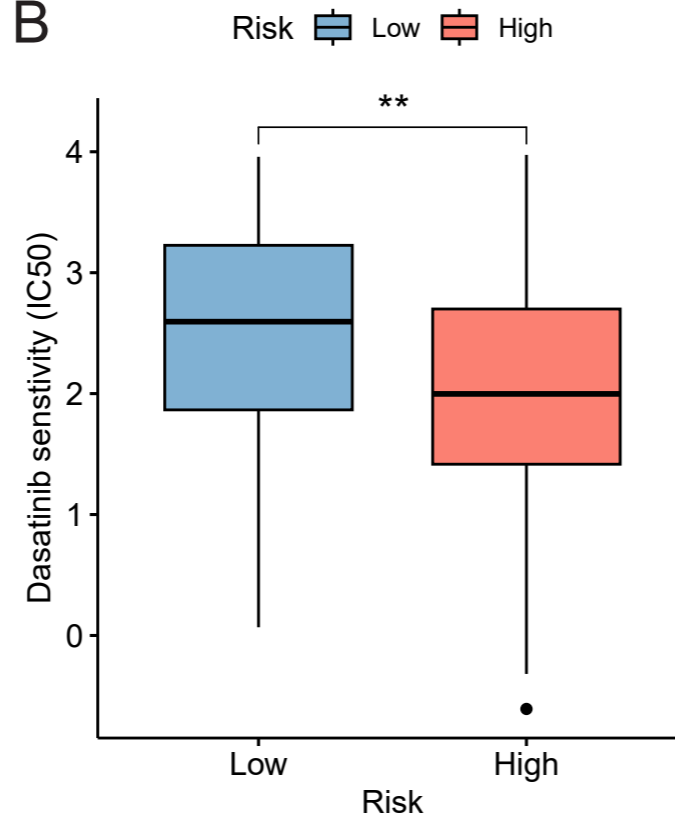

C

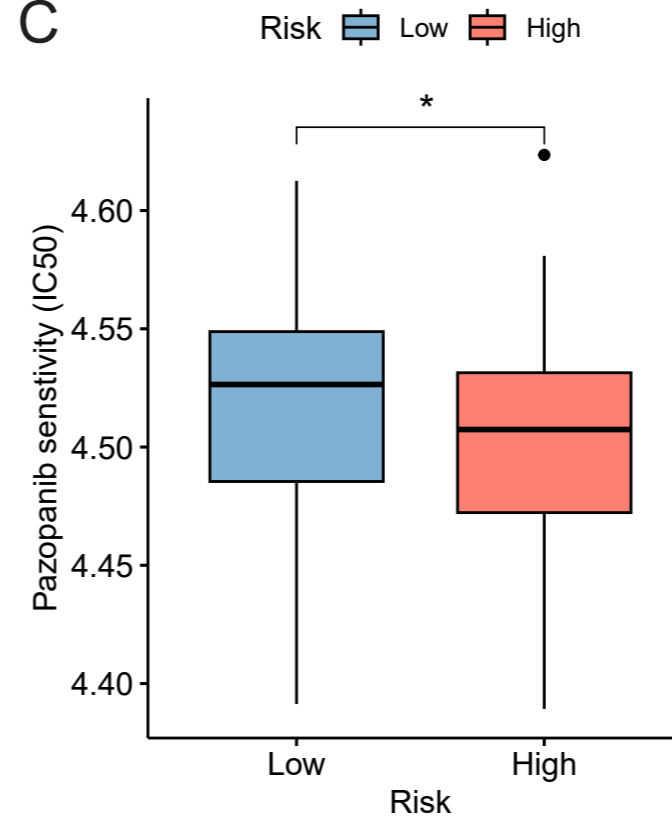

D

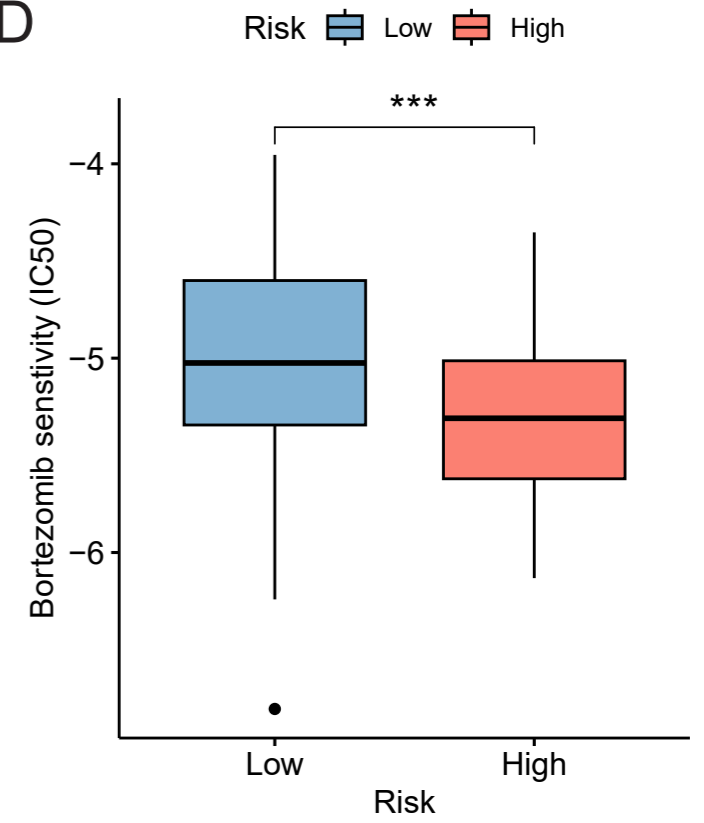

E

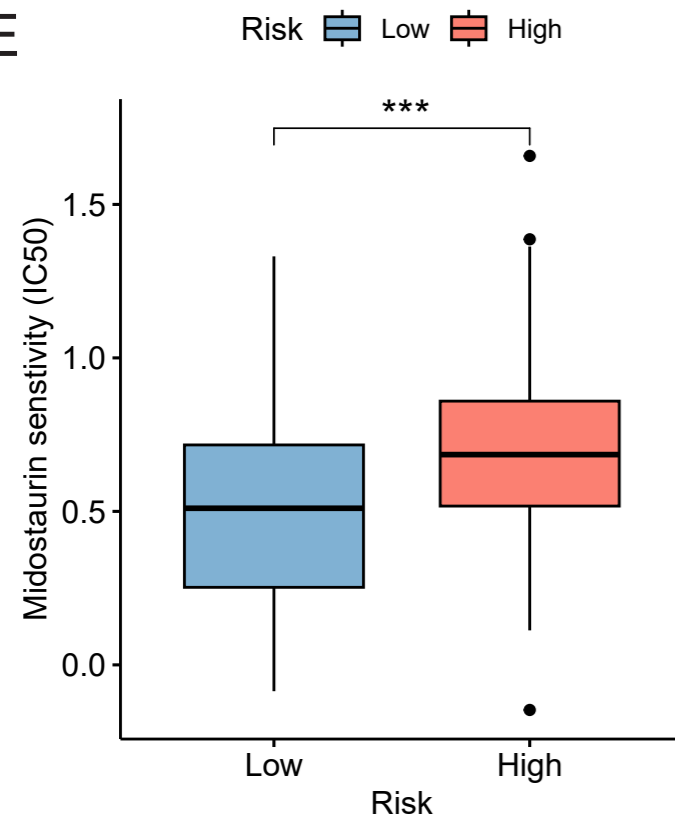

F

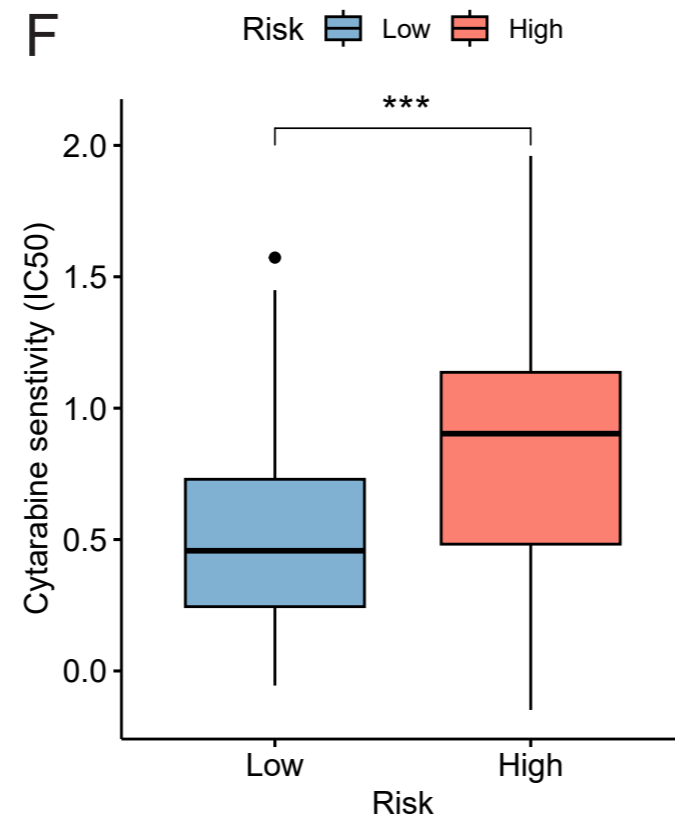

G

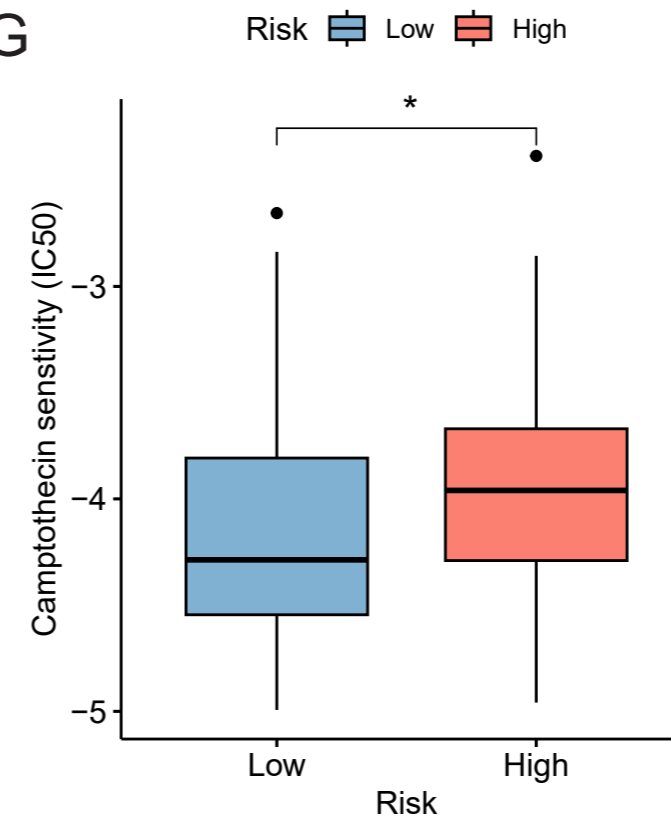

H

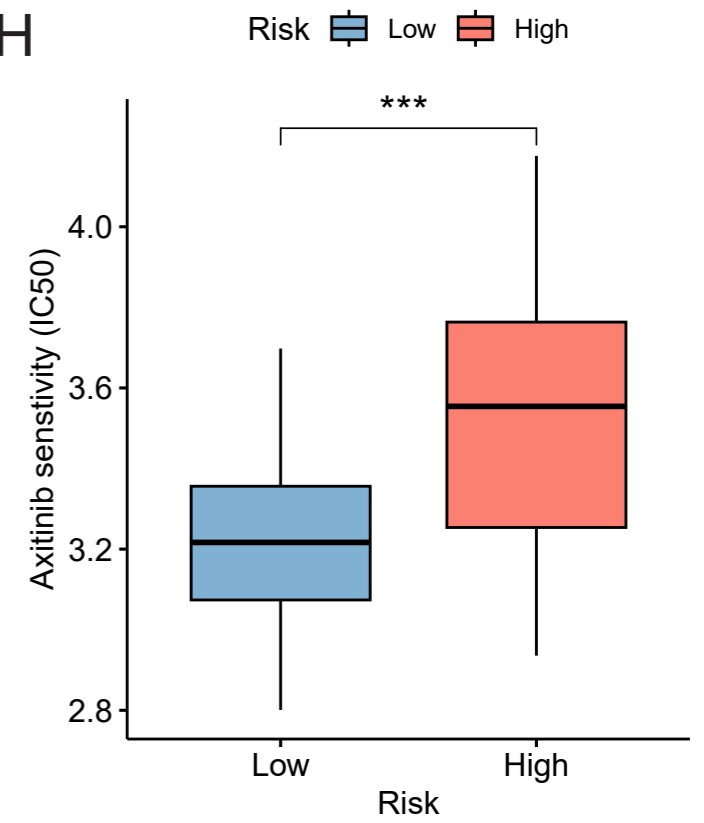

Supplement: Supplementary Materials — Figure S1: drug sensitivity analysis of the macrophage-related index. Sensitivity analysis for sorafenib (A), dasatinib (B), pazopanib (C), bortezomib (D), midostaurin (E), cytarabine (F), camptothecin (G), and axitinib (H) in patients between low and high macrophage-related index groups. Figure S2: biological analysis of macrophage-related index: (A, B) the results of GSVA enrichment analysis of Hallmark (A) and KEGG (B), (C) representative enriched GO terms of DEGs in macrophage-related index groups, (D) representative enriched KEGG terms of DEGs in macrophage-related index groups. Table S1 (abbreviation table): proprietary terms and their corresponding abbreviations. [file 5539065.f1.zip › Figure S1-revised (1).pdf]

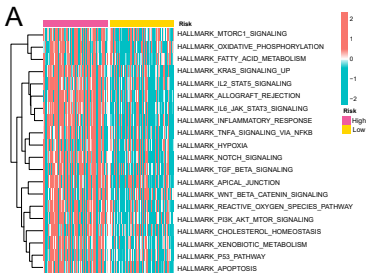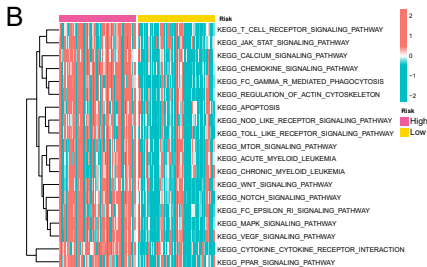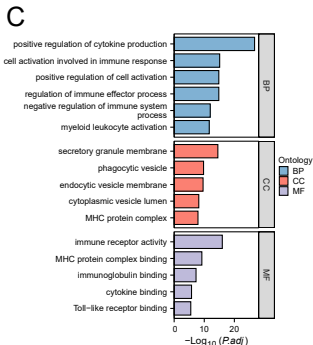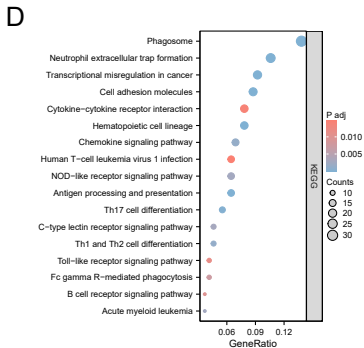

Supplement: Supplementary Materials — Figure S1: drug sensitivity analysis of the macrophage-related index. Sensitivity analysis for sorafenib (A), dasatinib (B), pazopanib (C), bortezomib (D), midostaurin (E), cytarabine (F), camptothecin (G), and axitinib (H) in patients between low and high macrophage-related index groups. Figure S2: biological analysis of macrophage-related index: (A, B) the results of GSVA enrichment analysis of Hallmark (A) and KEGG (B), (C) representative enriched GO terms of DEGs in macrophage-related index groups, (D) representative enriched KEGG terms of DEGs in macrophage-related index groups. Table S1 (abbreviation table): proprietary terms and their corresponding abbreviations. [file 5539065.f1.zip › Figure S2-revised (1).pdf]
